# Epistemology of the origin of cancer: a new paradigm

**DOI:** 10.1186/1471-2407-14-331

**Published:** 2014-05-10

**Authors:** Björn LDM Brücher, Ijaz S Jamall

**Affiliations:** 1 Theodor-Billroth-Academy®, Munich, Germany; 2 Theodor-Billroth-Academy®, Richmond, VA, USA; 3 Theodor-Billroth-Academy®, Sacramento, CA, USA; 4 INCORE, International Consortium of Research Excellence of the Theodor- Billroth-Academy®, Munich, Germany; 5 INCORE, International Consortium of Research Excellence of the Theodor- Billroth-Academy®, Richmond, Virginia, USA; 6 INCORE, International Consortium of Research Excellence of the Theodor- Billroth-Academy®, Sacramento, CA, USA; 7 Bon Secours Cancer Institute, Richmond, VA, USA; 8 Risk-Based Decisions, Inc., Sacramento, CA, USA

**Keywords:** Cancer, Paradigm, Inflammation, Fibrosis, Carcinogenesis, Tumor, Neoplasm

## Abstract

**Background:**

Carcinogenesis is widely thought to originate from somatic mutations and an inhibition of growth suppressors, followed by cell proliferation, tissue invasion, and risk of metastasis. Fewer than 10% of all cancers are hereditary; the ratio in gastric (1%), colorectal (3-5%) and breast (8%) cancers is even less. Cancers caused by infection are thought to constitute some 15% of the non-hereditary cancers. Those remaining, 70 to 80%, are called “sporadic,” because they are essentially of unknown etiology. We propose a new paradigm for the origin of the majority of cancers.

**Presentation of hypothesis:**

Our paradigm postulates that cancer originates following a sequence of events that include (1) a pathogenic stimulus (biological or chemical) followed by (2) chronic inflammation, from which develops (3) fibrosis with associated changes in the cellular microenvironment. From these changes a (4) pre-cancerous niche develops, which triggers the deployment of (5) a chronic stress escape strategy, and when this fails to resolve, (6) a transition of a normal cell to a cancer cell occurs. If we are correct, this paradigm would suggest that the majority of the findings in cancer genetics so far reported are either late events or are epiphenomena that occur after the appearance of the pre-cancerous niche.

**Testing the hypothesis:**

If, based on experimental and clinical findings presented here, this hypothesis is plausible, then the majority of findings in the genetics of cancer so far reported in the literature are late events or epiphenomena that could have occurred after the development of a PCN. Our model would make clear the need to establish preventive measures long before a cancer becomes clinically apparent. Future research should focus on the intermediate steps of our proposed sequence of events, which will enhance our understanding of the nature of carcinogenesis. Findings on inflammation and fibrosis would be given their warranted importance, with research in anticancer therapies focusing on suppressing the PCN state with very early intervention to detect and quantify any subclinical inflammatory change and to treat all levels of chronic inflammation and prevent fibrotic changes, and so avoid the transition from a normal cell to a cancer cell.

**Implication of the hypothesis:**

The paradigm proposed here, if proven, spells out a sequence of steps, one or more of which could be interdicted or modulated early in carcinogenesis to prevent or, at a minimum, slow down the progression of many cancers.

## Background

Cancer is a complex and heterogeneous set of diseases with no simple definition [1]. A century ago, tumor growth alone was considered the fundamental derangement, and tumors were classified and described in terms of their growth rates: (1) slow, (2) moderately rapid, and (3) rapid [2]. Today, carcinogenesis is thought to be triggered by mutations [3] and an inhibition of growth suppressors, which, in turn, gives rise to the cell proliferation, tissue invasion, and risk of metastasis [4].

### Mutation and polymorphism

Over the past several decades, the theory that somatic mutations are the primary trigger for carcinogenesis has become the predominant paradigm to explain the origin of most cancers. In fact, the German surgeon and cancer researcher, Karl-Heinrich Bauer (1928), on observing mutations in plants and animals, offered the then plausible biological explanation that cancers were likely caused by mutations [5]. Some rare cancers have indeed been shown to involve mutations, most notably the deoxyribonucleic acid (DNA) damage that ensues from exposure to non-lethal doses of ionizing radiation [6]. The Watson and Crick discovery, aided by Rosalyn Franklin’s X-ray diffraction study of DNA [7], achieved in large measure by “*theoretical conversation…little experimental activity*” [8], served to elucidate the three-dimensional structure of DNA [9] and gave credence to the concept that damage to DNA molecules can lead to cancer. Although some 50 years ago, Ashley stated that cancer may be the result of just 3 to 7 mutations [10], and since then, others have proposed different possible numbers of critical mutations [11,12], the number necessary to cause a normal cell to change to a cancer cell is not yet known. The clinical and laboratory evidence suggests that carcinogenesis requires more than mutations since, in order for a cancer to develop, the DNA repair mechanism would have to be absent, defective, or inefficient, as seen, for example, in children with Xeroderma pigmentosum [13]. Somatic mutations are increasingly questioned as drivers of carcinogenesis [14,15], and some cancers are not associated with any mutation [16,17]. Furthermore, the inactivation of tumor suppressor genes is also involved in the cell transformation process [18]. In this context, one group of researchers has suggested illuminating the process by comparing genomes among different species for example, those of a mouse or rat to those of the naked mole rat, which is resistant to cancer [19]. In recent years, the contribution of chronic inflammation to cell transformation has been revisited, although the mechanism of inflammation and its importance have yet to be elucidated [20]. Long thought to play a role in the development of cancer, inflammation is again under scrutiny, in light of recent data.

Until recently, the source of cancers was thought to be (1) hereditary, (2) infectious or (3) sporadic. Hereditary cancers occur in 5 to 10% of all cancers and in some 8% of breast and ovarian cancers, which are associated with genetic changes as BRCA1 or BRCA2 [21]; the equivalent figure for colorectal cancer is between 3 and 5%. Some 15% are thought to be caused by infection [22,23], a ratio perhaps misleading, as it is about 60% of gastric cancers and as high as 80% of hepatic cancer [24]. The remaining cancers (70-80%) are considered sporadic, a euphemism for “unknown cause”. Only 15% of sporadic cancers are traced to somatic mutations [25], but a carrier is not automatically afflicted, although his risk for the associated cancer may be greater than 50%. Intra-patient heterogeneity and variability have always hampered the search for uniform and effective therapies, and heterogeneity remains a huge impediment to assigning one origin to many different types of cancer.

Fully 99.9% of all mutations that occur within the coding regions of the genome are not understood, nor have they been investigated. Additionally, the number of mutated genes and mutations per cancer are, a small percentage of mutations in a coding region varies greatly [26]: 97% of mutations are single-base substitutions and about 3% are insertions or deletions. Furthermore, of the reported single-base mutations, 90.7% are missense changes, 7.6% are nonsense, and 1.7% involve splice sites located in non-translated regions that immediately follow a start or stop codon. The number of mutated genes varies, with a smaller number of somatic mutations observed in the population of younger patients with a cancer than that of older patients with the same cancer. The number of observed mutations varies among tissues of the source cancer: tissue of cancers with high rates of cell division, such as the colon, exhibit more mutations per cell than that of cancers in slowly dividing tissues, such as the brain [26,27].

The enormous variability of mutations, combined with the fact that more than half of these occur even before the cancer phenotype is established, leads to an elevated “*noise to signal*” ratio in the exon sequencing data [26,27]. Mutations are assumed to occur over long periods of time - even as long as several decades. Because of the long time frame, it is reasonable to assume that the data from sequencing vary greatly according to the time of sample collection. Investigation to understand mutations is of significant importance to understanding even more profound underlying biological processes.

Genetic polymorphism is also important for understanding the processes, as two or more different phenotypes may exist in the same individual. Biologists usually investigate certain point mutations in the genotype, such as single-nucleotide polymorphisms (SNPs) or variations in homologous DNA by restriction fragment length polymorphisms (RFLPs), with chromatography, chromosome cytology, or by exploiting genetic data. Neither the mechanisms nor the distribution of different polymorphisms among individual genes are well understood, although the latter is considered a major reason for the evolutionary disparity that survives natural selection [28]. Polymorphisms are necessary to understanding biology - including tumor biology - but are not be the key to solving cancer genomics.

The reasons why polymorphisms are not a viable route for unraveling cancer genomics are multiple: (1) We do not understand how polymorphisms reflect a disease or respond to a treatment, or even if they react in coordination with other polymorphisms in other genes. (2) On 23 July 2013, the number of SNPs published in the Single Nucleotide Polymorphism Database (dbSNP) was 62,676,337 [29]. (3) Human beings have 23 paired chromosomes (46 in each cell) and, according to data from the Human Genome Project, humans probably have 21,000 haploid coding genes with approximately 3.3 × 10^9^ base pairs [30]. (4) Chromosome 1 of the 46, with its 249,250,621 base pairs, has 4,401,091 variations [31]. (5) The mutation rate is estimated to be 10^-6^ to 10^-10^ in eukaryotes [32], a piece of data that could permit a calculation of the possible combinations. (6) However, the number of pseudogenes - about 13,000 [30] - and (7) the wide variation of transposable (mobile) genetic DNA sequences complicate such a calculation [33,34]. For example, *Alu* has about 50,000 active copies/genome, while another, LINE-1 (=long interspersed element 1), has 100. (8) To the best of our knowledge, mobile genetic elements - classified under CLASS I DNA transposons as LTRs (long terminal transposanable retroposons) and non-LTRs, such as long interspersed elements (=LINEs) and short interspersed elements (=SINEs), and CLASS II DNA transposons - account for more than 40% of the total genetic elements [35].

In addition to these eight reasons, we note that neither the genetic information nor the different cells alone influence biological processes [36]; the extracellular matrix (ECM) is essential for cellular differentiation and thus influences that differentiation directly, as well as providing stabilizing ligament fibroblasts [37]. Moreover, only 50% of patients with disseminated tumor cells and circulating tumor cells (CTCs) develop clinically evident metastatic cancer, and only 0.01% of disseminated cells and CTCs develop metastasis [38,39]. Even the fact that cancerous cells have been observed *in vitro* without inflammation or fibrosis does not account for the vast majority of cancers for which mutations cannot explain their development.

Normal cellular processes that damage DNA include the generation of reactive oxygen species (ROS), alkylation, depurination, and cytidine deamination [40]. The magnitude of DNA damage affected by normal cellular processes is enormous, estimated at approximately ten thousand depurinated sites generated per cell per day; an even greater number of alterations results from ROS [41,42]. This DNA damage is continuously monitored and repaired; over 130 DNA repair products have been identified [43]. In normal cells, DNA replication and chromosomal segregation are exceptionally accurate processes. Measurements of the mutagenesis of cells grown in culture yield values of approximately 2×10^-10^ single base substitutions per nucleotide in DNA per cell division, or 1×10^-7^ mutations/gene/cell division. An even lower number has been demonstrated in cultured stem cells [40,44]. Taking into account this very low frequency of mutation, the spontaneous mutation rate of normal cells seems insufficient to generate the large number of genetic alterations observed in human cancer cells. If a cancer arises in a single stem cell, then the spontaneous mutation rate would account for less than one mutation per tumor. That discrepancy led to a hypothesis, as yet unproven, of a “*mutator phenotype,*” which - by envoking genomic instability - might account for the greater number of somatic mutations observed [45].

These sobering considerations reflect the complexity of biological processes. We think it unlikely, logically and computationally, to find the needle - the origin of cancers - in this huge haystack. After depending on the somatic mutation paradigm for some 85 years, these considerations justify contemplating a paradigm shift. Biological processes as well as cell-cell communication and signaling are themselves a multidimensional musical opera in different acts, which are played differently by different symphony orchestras rather than by a soloist. Even the composition of the music, which is needed before it can be played, is not well understood.

**Figure 1:**
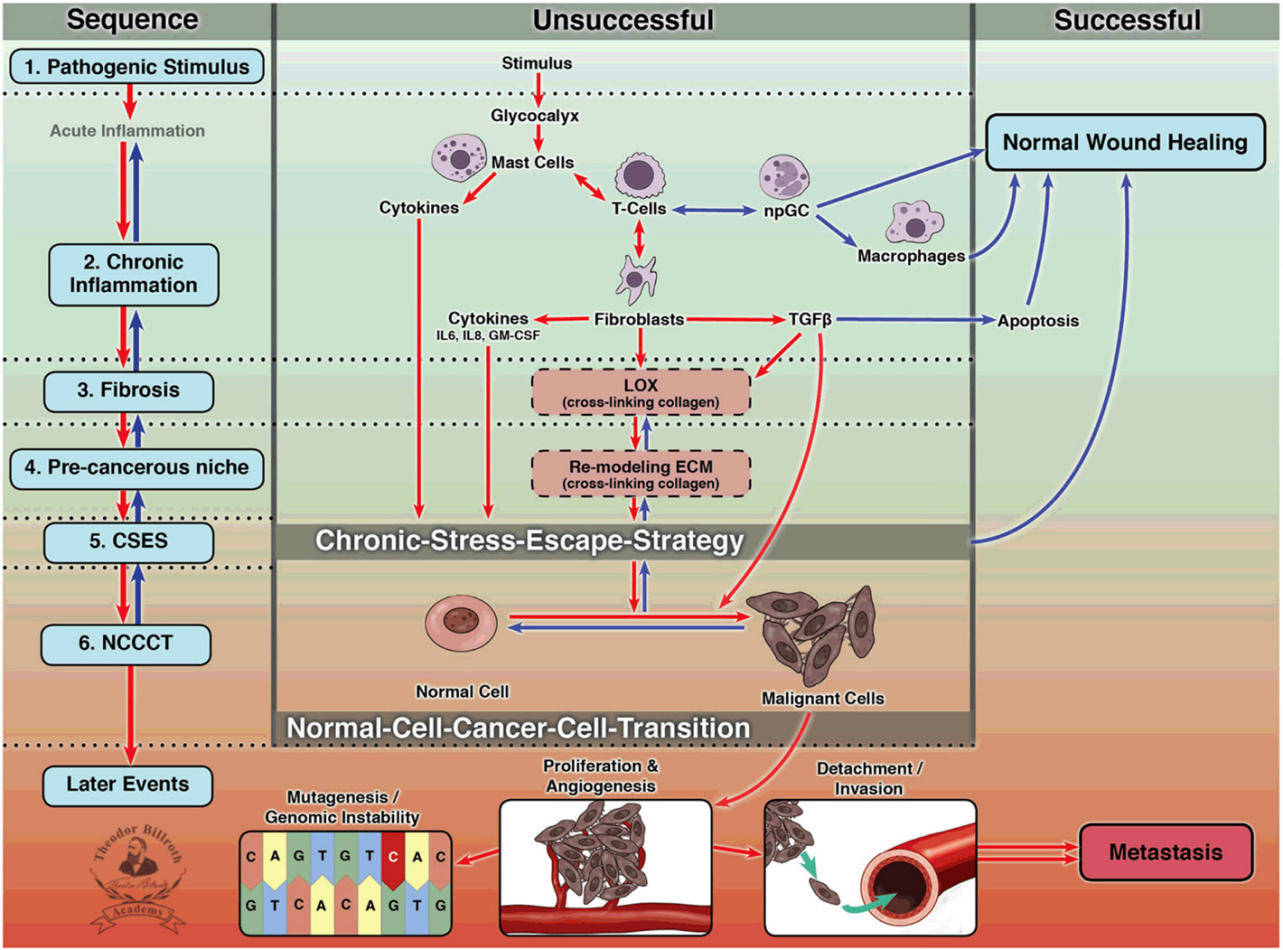
**Schematic drawing of “Epistemology of the Origin of Cancer”.** Abbreviations: CSES, chronic stress escape strategy; NCCCT, normal cell cancer cell transition; npGC, neutrophil Granulocyte; TGFβ, tumor growth factor beta; LOX, Lysyl oxidase; ECM, extracellular matrix.

We propose an alternate hypothesis for the origin of the majority of cancers. Our paradigm postulates that cancer originates following a sequence of events that include (1) a pathogenic stimulus (biological or chemical), followed by (2) subclinical chronic inflammation, from which develops (3) fibrosis with associated changes in the cellular microenvironment. From these changes, (4) a pre-cancerous niche (PCN) develops, which triggers (5) deployment of a chronic stress escape strategy (CSES) with (6) a normal cell-cancer cell transition (NCCCT) (Figure 1). In this paper, we justify our hypothesis by showing why it deserves consideration as the explanation for the genesis of most cancers.

## Presentation of the hypothesis

(1) * Pathogenic Stimulus*

The earliest information a cell receives is a pathogenic (biological or chemical) stimulus. The first receiver seems to play a major role in processing the stimulus. Chemical carcinogenesis is thought to be a two-step process: in the first step, called “initiation,” the carcinogen damages or binds to nuclear DNA; in the second step, referred to as “promotion,” some other chemical or physiologic event facilitates the aberrant growth that ultimately results in cancer. The classic example was reported by Yamigawa and Ichikawa in 1915, when they applied coal tar derivatives to rabbit ears and observed skin cancer [46]. Subsequent work showed that dermal application of several different polyaromatic hydrocarbons (PAHs), such as benzo[a]pyrene and benzo[a]anthracene, followed by a phorbol ester (a promoter), generated skin cancers in a dose-dependent manner. Over time, alkylating agents, such as sulfur mustard, ethylene dibromide, and many nitrosoamines, were included in the list of chemicals that could give rise to cancer, both in experimental animals and in humans. The list grew to include arsenic, hexavalent chromium, mycotoxins - notably aflatoxins - ionizing and ultraviolet radiation, cigarette smoke, and asbestos, to name the most egregious compounds linked to cancer. Phenotypes of cancer cells can be the result of mutations, i.e., changes in the nucleotide sequence of DNA, which accumulate as tumors progress. Such mutations can arise as a result of DNA damage or by the incorporation of non-complementary nucleotides during DNA replication. In the past decade or so, it has been postulated that a cancer must exhibit a “*mutator phenotype*” that leads to genomic instability, but whether or not the acquisition of a mutator phenotype is necessary for tumor progression remains unproven [45].

We have long known that nearly all cells are coated with a thin layer of glycoprotein and acidic material outside the plasma membrane, called the glycocalyx [47], which consists of polysaccharides covalently bonded to membrane proteins (90% glycoproteins and 10% glycolipids). The surface and size of the glycocalyx that coats biological membranes differ in their specific function. The glycocalyx in mammalian cells contains 5 classses of phylogenetically conserved molecules for adhesion: (1) immunoglobulins (2) integrins (3) cadherins (4) selectins, and (5) cell adhesion-molecules. Through these, the glycocalyx contacts the microfilament (cytoskeletal) system of the cells, couples with GTP-binding proteins of the cell membrane, and communicates between cells and their microenvironment. Other functions include protecting the cell and underlying tissues from dehydration or phagocytosis, providing adherence on the surfaces, acting against a pathogenic factors, interacting in cell-to-cell communication, and in vessels, housing vascular protective enzymes [48].

Due to its oligopolysacharide polymers and sialic acids, the glycocalyx surrounding mammalian cells is negatively charged. The resulting electrostatic repulsion is thought to be important in protecting cells from non-specific adhesions [49] and, reportedly, that “*specific lock-and-key-type adhesion molecules overcome this repellent force*” [50]. Downregulation of the repelling components of the glycocalyx in oligodendrocytes brings extracellular surfaces separated by long distances closer together, a finding that could explain the way changes in pH or ion concentrations seem to influence myelin destabilization in multiple sclerosis [51]. Moreover, ROS cause proteinuria by modulating the barrier function of the glomeruli endothelial glycocalyx [52]. Disruption of the glycocalyx in vascular tissue results in inflammation and thrombosis, and is under investigation in the search for new cardiovascular drugs [53]. We think that because it receives information first from a pathogenic stimulus the glycocalyx deserves greater emphasis in the effort to elucidate its significance in cancer.

(2) *Chronic inflammation*

Some 230 years ago, the British physician, Sir Percival Pott, reported a high incidence of scrotal cancers in chimney sweeps, suggesting that irritation by soot led to a chronic inflammation of the scrotum and that, in turn, resulted in the scrotal cancers in this cohort [54]. Later, in 1863, Virchow observed leukocytes in neoplastic tissue [55], indicative of inflammation, but he could not determine whether the inflammation was a cause or an effect of the accompanying neoplasia. John Chalmers da Costa reported two cases of squamous cell carcinoma within chronic ulcers and noted, “[it is] *believed, that cancer may arise* … *in an area of chronic inflammation*” [56]. As mentioned above, in the early 20^th^ century, Yamagiwa and Ishikawa repeatedly applied coal tar to rabbit ears and observed the resultant tumor growth, which was preceded by chronic inflammation [46]. William Gye used acriflavine, other antiseptics, and heat treatment to inactivate filtrates from the *Rous* sarcoma, which were free of tumor cells, and demonstrated that these filtrates gave rise to chronic inflammation before the onset of the cancer [57].

All organisms attempt to resolve the disruption of cells and tissues caused by inflammation, a complex and multifactorial process that usually results in wound healing. Persistent acute inflammation due to non-degradable pathogenic stimuli such as a viral or bacterial infection, a persistent foreign body, or an autoimmune reaction results in unresolved wound healing with consequent chronic inflammation. Between acute and chronic inflammation lye a wide range of overlapping processes; the kind of inflammation found at the midway point of that range is often referred to as sub-acute inflammation [1]. In addition to the differences between acute and chronic inflammation, a difference between local and systemic wound responses, in terms of inter-tissue and organ communications, also exists [58]. Modulation of cell interacting junctions is maintained for epithelial integrity and, in particular, desmosomes, connexins, and adhesion complexes are downregulated at the wound edge [59,60]. The major cells involved are mononuclear: monocytes, lymphocytes, plasma cells, fibroblasts, and, especially, mast cells (MCs). Paul Ehrlich, in 1878, first described MCs in detail [61]; more recently, they have been reported as a component of the tumor microenvironment *reviewed in *[62]. MCs are thus a significant communication link between a pathogenic stimulus, the glycocalyx, and the cell stroma directly and/or via fibroblasts. MCs can be activated directly by a pathogen or indirectly by binding to such receptors as the high-affinity immunoglobulin E (IgE) receptor FcϵRI, as well as through pattern recognition receptors (PRRs), e.g., toll-like receptors (TLRs) [63,64] and G-protein-coupled receptors (GPCRs) [63]. MCs present native protein antigens to CD4+ T-cells and act as antigen-presenting cells (APC); both cell types influence each other in an antigen-dependent manner [65]. CD4+ T-cell populations, with their regulatory interactions, play a role in the host response to pathogenic stimuli [66]. Contact-mediated activation of endothelial cells by T-cells involving a ligand such as CD40 may serve as one mechanism for the continuous progression of inflammatory diseases in atherosclerosis and rheumatoid arthritis [67]. Immune cells and their cytokines have been reported to be associated with carcinogenesis and T-cell-infiltrating tumors such as ovarian, breast, prostate, renal, esophageal, colorectal carcinomas, and melanomas, all of which have been correlated with patient outcome [68-74]. Stromal cell-related cytokines of inflammation such as tumor necrosis factor alpha (TNF-α) activate the nuclear factor kappa-light-chain-enhancer of activated B cells (NF-κB), which plays an important role - not completely understood - in carcinogenesis [75,76]. Inflammation “associated” cells, as well as the tumor microenvironment, interacts with all different types of immune cells [20,77], and MCs effectively communicate among vascular, nerve, and immune system cells [78].

To date, some 15% of all human cancers are reported to originate from infectious disease [22,23]. However, the majority of cancers arises spontaneously and is attributed to an unknown etiology*.* Although formally designated as “unknown etiology,” under the existing paradigm an accumulation of a number of somatic mutations greater than some threshold not yet defined is considered to be the principal triggering factor. Chronic inflammation is known to lead to derangement in signaling processes and to a local microenvironment described as lying somewhere between pre-cancerous stromal cells and cancer cells [79], even as the details of the steps in the transformation to a cancer cell are incompletely understood [80]. Earlier findings [81], recently revisited [82], demonstrated that wound healing leads to a microenvironment similar to the hospital-observed stroma of tumors. The tumors were compared to wounds that do not heal [83]. A complex biological and immunological process [84] leads to all of the five signs of cancer first noted by Celsus and Galen [85]: *dolor* (pain), *calor* (heat), *rubor* (redness), *tumor* (swelling) and *function laesa* (loss of function).

It has been stated that “*the direct link between pathogen-specific gene products and a stereotypical altered host response key to disease development is missing*” [86]. Observations in epidemiology and laboratory research have generated sufficient evidence that chronic inflammation evokes an increased susceptibility to cancer [87]. The association of chronic inflammation and cancer makes the fact that a low-dose aspirin regimen, known to suppress prostaglandin-H2-synthase (COX-1, COX-2), could have an anticancer effect in colorectal cancer [88]. We have no data on the prevalence of “silent” inflammation, as it is often low-level and sub-clinical, but we do know that a weakened immune system may facilitate the initiation of tumor growth [89]. Eliminating the triggering event for infection or inflammation typically results in healing and tissue repair. If the infection or consequent inflammation is not completely resolved, it simmers as a chronic inflammatory condition [90], setting up one of the pre-conditions for transforming normal cell to cancerous cells.

The primary mediators of cells involved in inflammation are IFN-γ (equivalent to macrophage-activating factor), other cytokines, growth factors, ROS (released by macrophages), and hydrolytic enzymes. ROS are toxic for the organism and the tissue, and both are usually protected against ROS by alpha-1-microglobulin, superoxide dismutases (SOD), catalases, lactoperoxidases, glutathione peroxidases, and peroxiredoxins [91]. Exogenous ROS can come from pollutants, tobacco, smoke, xenobiotics, or radiation; endogenous ROS are produced intracellularlily through multiple mechanisms. Depending on the cell and tissue, the major ROS sources are the dedicated producers: NADPH oxidase, (NOX) complexes (7 distinct isoforms) in cell membranes, mitochondria, peroxisomes, and the endoplasmic reticulum [92]. The resulting oxidative stress affects not only cells but also the ECM, which is thought to enjoy less antioxidant capacity than do cells: Madsen and Sahai stated that the *“cytoskeleton of a typical epithelial cell and many cancer cells is not adapted to withstand stresses*” and that the microenvironment of acute inflammation differs significantly from that of chronic inflammation [93]. Additionally, the proteins of connexins, Cx43 and Cx32, are synthesized and integrated into the cell membranes of MCs [94], monocytes [95], leukocytes [96], and *Kupffer* cells [97]. They have also been found in cells associated with brain tumors [98], *reviewed in *[99]. Thus, cell types such as those of the brain and immune system can communicate with their microenvironment via expressed connexins.

Cancer has been linked to various pathogens, including the *Epstein-Barr virus* (*EBV*) in *Burkitt’s* lymphoma and nasopharyngeal carcinomas [100] and *human papilloma virus* (*HPV*) in cervical cancer [101]. In 2005, the *Nobel* Prize honored the discovery that infection by *Helicobacter pylori* (*H. pylori*) leads to inflammation, gastritis, and peptic ulcer [102]. The fact that *H. pylori* increases the risk of gastric cancer is widely accepted [103]. When it infects, *H. pylori* attaches to cell-cell interfaces and the bacterium changes it shape, adhering to the cell and secreting outer membrane vesicles [104]. It has been shown that the extent of “*loss or dysfunction of E-cadherin was proportional to the migratory behavior of tumor cells and its metastatic potential*” [104-106]. Loss of E-cadherin is associated with loss of cell-cell adherens and increased epithelial permeability. Within 48 hours after *H. pylori* infection, a significant proportion of E-cadherin was found in small vesicles within the cell [107]; furthermore, vacuolating cytotoxin VacA from *H. pylori* enhanced the association of intracellular *H. pylori* vesicles containing lipopolysaccharide [108]. We assume these are the effects of the chronic inflammatory processes because, according to the Kuehn and Kesty review [109], so-called membrane vesicles of bacteria contain not just lipopolysacharides, but also chromosomal, plasmid, and phage DNA [110-112].

Why do all chronic inflammations not result in cancer? If chronic inflammation, per se, were a sentinel event in the transformation of a normal cell to a cancer cell, one would expect a high incidence of cancer in patients with chronic arthritis, but that is not evident. The nature of the inflammation that can facilitate the development of cancer, and of that that does not, is as yet unexplained. Patients with rheumatoid arthritis have a greater risk than non-arthritic patients for lymphoma, melanoma, and lung cancer, but not of colon cancer or breast cancer [113]. We do know, however, that severe pneumonitis associated with either bacterial pneumonia or tuberculosis resolves completely with treatment, whereas inflammation associated with *H. pylori* can result in gastric cancer in about 60% of cases, and with hepatitis B or C, in liver cancer in as many as 80% of chronic infections [24]. Perhaps the distinctive feature in the inflammation that promotes the conversion of a normal cell to a cancerous one is its ability to trigger the onset of fibrosis. For example, pulmonary mesothelioma, known to be caused by exposure to asbestos, generally presents decades after exposure. Its appearance is always preceded by inflammation and by severe fibrosis [114]. No increase in the number somatic mutations has been associated with asbestos carcinogenesis. In a mouse model of experimental hepatocellular carcinoma (HCC), injection of a single dose of an initiator such as diethylnitrosamine (DEN), followed by repeated sub-toxic doses of carbon tetrachloride (promotor), resulted in both inflammation and fibrosis, as well as a 100% incidence of HCC that mimicked the human disease [115]. Furthermore, only recently, ultraviolet radiation-induced inflammation has been demonstrated to promote angiotropism and metastasis in melanoma; blocking the inflammation alone markedly reduced the incidence of metastasis [116,117]. Patients with chronic inflammatory diseases can develop cancer after variable latency periods. For example, a long-term follow-up of patients with oral pre-cancerous lesions demonstrated an increased risk for oral cancers after 5 and 10 years of about 5% and 10%, respectively [118].

(3) *Fibrosis and changes in the microenvironment*

Since chronic scars were first linked to the onset of cancer, well over 100 years ago, chronic inflammation has been associated with fibrosis [119]; Hepatitis B and C infections are related to hepatocellular carcinoma (HCC) in patients who first develop liver fibrosis [120]. A recent review of cell-cell communication between MCs and fibroblasts states, “*The remodeling phase of inflammation may explain chronic fibrosis*”; preventing the accumulation of MCs and their interference of fibroblast activation via connexins may offer a new approach to prevent excess scarring [121]. The process of fibrogenesis, an integral part of wound healing as the organism tries to resolve chronic inflammation, is governed by three factors: continuous stimulus, an imbalance of collagen synthesis versus degradation, and a decrease in the activity of the degradative enzymes involved in removing collagen [122]. One key enzyme for the permanent cross-linking of single triple-helix collagen molecules (multiple tropocollagen molecules) is the copper (Cu)-dependent amine oxidase, lysyl oxidase (LOX), discovered by Pinnell and Martin in 1968 [123].

LOX is an extracellular amine oxidase that catalyzes the covalent crosslinking of ECM fibers. Collagen I, a component of both desmoplastic tumor stroma and organ fibrosis is a major substrate for LOX and has been shown to be a key component of both primary and metastatic tumor microenvironments [124,125]. Elevated levels of procollagen I, a collagen I precursor, have been found in the serum of patients with recurrent breast cancer [126]. They also have been shown to drive the activation of dormant D2.OR cells seeded to the lung [127]. LOX activity was reported to be greater in human breast cancer than in normal tissues [128], a finding that suggests that LOX plays a key role in creating the cellular microenvironment necessary for a pre-cancerous niche (PCN), one of the prerequisites for the induction of cancer. LOX overexpression is found in myofibroblasts and myoepithelial cells around *in situ* tumors and at the invasion front of infiltrating breast cancers [129]. It was shown to be essential for hypoxia-induced metastasis [130] and, more recently, it has been rather elegantly demonstrated that targeting LOX prevents both fibrosis and metastatic colonization [131]. Furthermore, LOX modulates the ECM and also cell migration and growth [132]. Studies in the blind mole rat, *Spalax*, revealed that the fibroblasts in this species suppress the growth of human cancer cells *in vitro *[133] and decrease the activity of hyaluronan synthase 2 [134]. This species was also resistant to chemical carcinogenesis. These data constitute evidence that fibrosis is necessary for establishing the PCN stage, an intermediate stage on the path from a normal cell to a cancer cell. Additionally, it has been shown that necrotic wounds induced in *Spalax* by chemical carcinogens heal with no sign of malignancy [133], a finding that supports our hypothesis that the PCN stage is key to the transformation of a normal cell to a cancer cell.

Some of the LOX findings are paradoxical [135]; we assume the paradoxes are due to the fact that early investigators did not differentiate among the different LOX isoforms. That LOX was expressed in 79% of human breast cancers revealed the attenuated metastasis of human breast cancer cells by a downregulation of adhesion kinase and the paxillin-signaling pathway [128,136]. SNPs in the LOX-like protein 4 were reported in patients with endometriosis, a semi-malignant tumor [137]. LOX overexpression can be found in myofibroblasts and myoepithelial cells around *in situ* tumors and at the invasion front of infiltrating breast cancers [129]. Further, LOX is downregulated in squamous cell skin carcinomas [138], head and neck cancers [139], upper gastrointestinal carcinomas [140-142], and renal carcinomas [143]. LOX expression was shown to be upregulated only in the presence of fibroblasts, suggesting that stromal fibroblasts directly influence LOX regulation [144]. This finding is concordant with one previously described, that targeting LOX prevents fibrosis and metastatic colonization [131]. The ECM itself provides biochemical and physical signaling to modulate and sustain surrounding tissue and cells (tumor microenvironment). LOX induction is mediated by both tumor growth factor beta (TGFβ-) and Smad and non-Smad JNK/AP-1 signaling pathways; it has been shown *in vitro* that LOX expression is blocked by “*TGFβ inhibitors as well as by inhibitors of the canonical Smad2, -3, and -4 signaling and non-Smad JNK/AP-1 signaling pathways*”. [145] This regulation of LOX is mediated in endothelial cells by such adhesion molecules as P-selectin, vascular cell adhesion molecule (VCAM-1), intracellular adhesion molecule (ICAM-1), and monocyte chemotactic protein (MCP-1) [146]. Furthermore, Cx43 expression is paralleled closely by that of adhesion markers such as VCAM-1, ICAM-1, and MCP-1 [147].

A number of reasons could explain the discrepancies reported of the down- and upregulation in LOX. Among these are the following: (1) Biomarkers, such as tissue inhibitors exhibit different levels of expression in tumor tissue compared to the tumor invasion zone or normal tissue. For example, Kopitz *et al*. investigated tissue inhibitor of metalloproteinase 1 (TIMP-1) in liver metastasis with reported significantly different expression levels in (a) tumor tissue, (b) invasion zone tissue, and (c) normal tissue [148]. (2) Remodeled ECM (pre-cancerous niche - PCN) as well as normal-cell-to-cancer-cell transitions were in different stages of completion. The LOX concentrations that differed according to the type of tumor may also reflect that both re-modeled ECM (pre-cancerous niche - PCN) and normal-cell-to-cancer-cell transitions were encountered in different stages of completion, and thus the resulting expression levels were different. (3) The finding of LOX upregulation in the invasion zone of breast cancer tissue has been reported [129]. (4) Researchers on LOX usually do not differentiate among the known isoforms of the enzyme (LOX, LOX1, LOX2, LOX3 and LOX4), although - even though they catalyze the same biochemical reaction - they differ in their amino acid sequence [149,150]. The LOX isoforms are encoded by different genes (on chromosomes 5, 15, 8, 2, and 10, respectively), have different molecular weights, differ in their percentage of similarities to the LOX domain (100, 85, 58, 65, and 62, respectively), and exhibit different protein sizes as well as different tissues, depending on their mRNA expression rates [151]. Moreover, LOX isoenzymes are expressed differently in different tissues [152]. (5) Different methodological approaches and protocols for measuring LOX could account for some of the reported differences. These five factors might explain some of the paradoxical findings reported for LOX.

The assumption that fibrosis is a necessary and thus a key step in the sequence of events preceding the transformation of normal cells to cancer cells is supported by the following evidence: (1) The presence of fibrosis is reported to increase the risk of acquiring cancer [153]. (2) Fibrosis with chronic inflammation is reported with a number of pre-cancerous lesions, e.g., actinic keratosis, *Crohn’s* disease, and *Barrett’s* metaplasia [154-156]. (3) Ongoing fibrosis, with fibrotic foci, has been observed in postmortem pancreatic cancer specimens [157]. (4) In cancer-resistant species such as the blind mole rat, *Spalax*, fibroblasts suppress the growth of cancers as well as the activity of hyaluronic synthase [133,134]. (5) In mice, chronic low-grade systemic inflammation leads to architectural changes that permit a mild level of alveolar macrophage infiltration [158]. (6) One of the features of oral submucosal fibrosis (OSF), a pre-cancerous condition, is chronic inflammation of the buccal mucosa accompanied by a progressive sub-epithelial fibrotic disorder [159].

(4) * Pre-cancerous niche and (5) Chronic-Stress-Escape-Strategy (CSES)*

The microenvironment of an acute inflammatory condition differs significantly from that of chronic inflammation, in which the host cannot eliminate the offending agent (a microorganism, a disease, or a toxin) because the “*cytoskeleton of a typical epithelial cell and many cancer cells is not adapted to withstand stresses*” [93]. Pathogenic stimuli induce chronic inflammation that, in turn, remodels the microenvironment, which itself develops fibrosis. This leads to a modulation of the ECM that, following exposure to chronic stress, may promote the formation of a pre-cancerous niche (PCN). Findings in the *Tasmanian Devil*, with its contagious cancer, led to an allograft theory [160]. Other authors have suggested that the near 100% mortality in this species was caused by the transmitted clonal tumor through downregulation of major histocompatibility complex (MHC) molecules [161], and they proposed an immunological escape strategy [162,163]. In an organism, the pathogenic stimulus, the chronic inflammation, and the fibrosis, which lead to a pre-cancerous niche, become a “vicious circle” thought to be resolved through a chronic-stress escape strategy (CSES). Histopathological investigations of 549 gastric ulcer patients revealed that about 70% of the lesions presented intestinal metaplasia within the regenerative epithelium, where chronic inflammation was considered the precursor of a pre-cancerous lesion [164]. We propose that chronic inflammation, with chronic TGFβ induction, serves to sustain a persistent stress in the cells of the host tissue. Furthermore, the distinction between the inflammation that promotes the development of a normal cell and that for a cancerous one lies in the ability of the inflammation to cause the onset of fibrosis. Asbestos leads to pulmonary mesothelioma decades after the exposure reveals fibrosis and, although no increase in somatic mutations has been reported in asbestos caused carcinogenesis, chronic inflammation has been observed in every instance of asbestos-induced mesothelioma [114]. These differences, in light of the proposed paradigm, are the duration of exposure to the pathogenic stimulus which reflects the importance of chronic inflammation and fibrosis in carcinogenesis.

The continuous release of TGFβ that is triggered by chronic inflammation has many effects: (1) TGFβ represses E-cadherin and occludin, increasing the adherens junction disassembly [165]. Inhibiting TGFβ receptor type-I has been shown to decrease its invasiveness [166]. (2) TGFβ induces miR21, a key regulator of mesenchymal phenotype transition [167], but increased levels also have been observed in early chronic fibrosis in COPD patients [168]. (3) TGFβ activates protein kinase B (AKT or PKB) through phosphoinositide-3 kinase (PI3K) [169], activating the mechanistic targets of rapamycin complex 1 (mTORC1) and mTORC2 [170]. Furthermore, TORC activates the translation of proteins important for cell growth and development, and the PI3K/TmTORC1 pathway has recently been shown both essential for cancer-associated inflammation [171]. (4) LOX and matrix metalloproteinase (MMPs) are induced by TGFβ [172], and (5) LOX activates PI3K [173]. (6) The phosphorylation of glycogen synthase kinase-3beta (GSK3beta) by AKT stabilizes SNAIL [174], which leads to an increase of TGFβ-induced SNAIL [175]. (7) SNAIL stability and activity, furthermore, are activated by LOX [176]. (8) TGFβ effects the dissociation of the long isoform of p120 from the membrane and its accumulation in the cytoplasm [177] and *Figure two B* in [178].

The chronic release of TGFβ and the continuous LOX activation trigger an accumulation of p120 in the cytoplasm, inducing remodeling of the ECM, which forms the pre-cancerous niche. This process may be seen as *the* starting point for the chronic-stress escape strategy. The p120 accumulation stimulates Cdc42 - a cell-division control protein and a member of the family of Rho small guanosine triphosphatases (GTPases) - and activates Ras-related C3 botulinum toxin substrate 1 (Rac1), decreasing thereby E-cadherin [179,180], microtubule polymerization [181], and integrin clustering [182]. Thus, the contact to the basal membrane is destabilized [183], promoting cell migration. In addition, p120 suppresses Rho activity by binding to exchange factor Vav2 and, in so doing, activates Rac1 [177]. As adherens junctions are regulated by Rho GTPases, suppressing Rho destabilizes the adherens junctions, increasing the dysregulation in the formation of cell-cell complexes. When microM antisense oligonucleotide was challenged by p120, after 4 h a decrease of 50% in the ratio of *in vitro* LOX cells in mitosis was observed and, after 8 to 72 h, as much as 70% [184]. These findings, together with the increase in both p120 and LOX activity, may indicate a p120 effect with an additional increase of LOX. SNAIL itself results in a decrease of E-cadherin [185,186], occludins [187], claudins [186,187], desmoplakin, and plakoglobin [188], and an increase in MMPs [189], fibronectin and vimentin [189], twist-related protein 1 (TWIST), zinc finger E-box-binding homeobox 1 (ZEB1), and ZEB2 [190]. With these cell interactions and communication mechanisms, all necessary conditions for cell transition have been accounted for: the formation of cell-cell complexes are deregulated, the stability of adherens junctions decreased, and the apical-basal polarity and re-organization of the cytoskeletal architecture lost.

(6) * Normal Cell-Cancer Cell Transition (NCCCT)*

The transition from one cell function to another, as well as the transition of one cell type to another seems to be a routine event rather than a rare one. Embryological studies have shown that the complex-building pancreatic homeodomain protein (PDX1) with pre-B-cell leukemia transcription factor 1 (PBX1) and the PBX-related homeobox gene MRG1 (MEIS2) results in building a multimeric complex which then switches the nature of its transcriptional activity in exocrine versus endocrine cells [191,192]. Additionally, it has been shown that an epithelial mesenchymal transition (EMT) in embryogenesis/morphogenesis acts in a direction opposite to that of a mesenchymal-epithelial transition (MET) [193]. Furthermore, EMT can induce non-cancer stem cells to become cancer stem cells [194,195].

Armin Braun recognized some 60 years ago that a gram negative bacterium *Agrobacterium tumefaciens* (*A. tumefaciens*) could initiate the *in vitro* transformation of normal plant cells into tumor cells; he showed that transformation occurs in a short time period, resulting in tumor cells with slower growth and less progression [196-198]. Ivo Zaenen *et al.* revealed, and Mary-Ann Chilton’s group subsequently proved, that a small DNA plasmid within *A. tumefaciens* was responsible for the transformation [199]: tumor inducing DNA (Ti-DNA), after infection, was integrated into the plant genome in tobacco plants [200]. Chilton also showed that Braun’s findings were based on the same principle: although the T-DNA from the *A. tumefaciens* Ti-plasmids was not at first detected [201], it was later proven to be in the nuclear DNA fraction of crown-gall tumors [202]. More evidence comes from research on mesothelial cells. In 1966, Eskeland, based on silver-staining electron microscopy studies, first suggested that injured or destroyed mesothelial cells are replaced in location and function by free-floating “peritoneal macrophages,” which are transformed from their original role to that of mesothelial cells [203,204]. This hypothesis was supported by further microscopy and electron microscopy studies from the same group [205,206] and by the later findings of Ryan and Watters [207,208]. As a consequence of a pathogenic stimulus such as inflammation or wound healing, EMT can change MCs into cells with mesenchymal or epithelial characteristics [122]. Xin reported supportive findings in prostate cancer that “*the cells of origin of cancer are the cells within tissues that serve as the target for transformation*” [209]. Similarly, studies in which Cx43 was knocked out to inhibit cell transition in corneal cells in vivo have shown that multifactorial regulated cell transition is influenced by cell-cell communication [210]. There is further evidence that a decrease in cell-cell adhesion is crucial for cell transition [211].

Because, under special circumstances, one type of human cell can transition to another, proposing that a normal cell transition to a cancer cell as one important sequence in carcinogenesis is justified. Additionally, evidence has been presented that a pathogenic stimulus gives rise to a molecular link of host immune response and genotoxic events, followed by inflammation also associated with carcinogenesis [212]. We propose that the observations in both the plant and animal kingdoms described above, taken together with the discovery of *H. pylori*, the finding that *EBV* can transform lymphocytes into cancer [213], and the identification of *HPV* 16 DNA [214] and *HPV* 18 [215] in cervical cancers (*HPV* infection is a precondition for about 75% of human cervical cancers) further support our hypothesis. EMT and MET were described as necessary for tissue repair and for migration, invasion, and metastasis [193]. We assume, in contrast, that - after a latency period in the CSES - a PCN results from chronic inflammation and fibrosis, and those conditions lead to a NCCCT.

To the extent that the above discussion proves the principle that chronic inflammation, including sub-clinical inflammation, can - after a latency period in the PCN stage - induce the a transformation of a normal cell to a cancer cell, finding biomarkers to define this sequence of events is important. The chronic inflammation and the fibrotic changes, including perhaps LOX activity, could explain the considerable aggression of many cancer cells, once transformed.

## Testing the hypothesis

We have described a new paradigm for the origin of the majority of cancers, based on observations and experimental findings in plants, animals, and humans. The paradigm postulates that most cancers originate from a stimulus and are followed by chronic inflammation, fibrosis, and a change in the tissue microenvironment that leads to a pre-cancerous niche (PCN). The organism responds with a chronic stress escape strategy (CSES), which, if not completely resolved, can induce a normal cell-cancer cell transition (NCCCT) (Figure 1).

If, based on experimental and clinical findings presented here, this hypothesis is plausible, then the majority of findings in the genetics of cancer so far reported in the literature are late events or epiphenomena that could have occurred after the development of a PCN. Our model would make clear the need to establish preventive measures long before a cancer becomes clinically apparent. Future research should focus on the intermediate steps of our proposed sequence of events, which will enhance our understanding of the nature of carcinogenesis. Findings on inflammation and fibrosis would be given their warranted importance, with research in anticancer therapies focusing on suppressing the PCN state with very early intervention to detect and quantify any subclinical inflammatory change and to treat all levels of chronic inflammation and prevent fibrotic changes, and so avoid the transition from a normal cell to a cancer cell.

## Implication of the hypothesis

We suggest that the majority of findings reported on the genetics of cancer are either late events or epiphenomena and that the different observations from basic and clinical research, combined with those from the plant, animal, and human world, justify our hypothesis. The development of cancer traces the following pathway: 1) pathogenic stimulus, 2) chronic inflammation, 3) fibrosis, 4) changes in the cellular microenvironment that result in a pre-cancerous niche, 5) deployment of a chronic-stress escape strategy, and 6) a transition from normal cell to cancer cell. The paradigm proposed here, if proven, spells out a sequence of steps, one or more of which could be interdicted or modulated early in carcinogenesis to prevent or, at a minimum, slow down the progression of many cancers.

## Abbreviations

Akt: Protein kinase B; APC: Antigen presenting cell; BRCA1: Breast cancer 1, early onset; BRCA2: Breast cancer 2, early onset; COX-1: Cyclooxygenase-1 (=Prostaglandin G/H synthetase 1); COX-2: Cyclooxygenase-2 (=Prostaglandin G/H synthetase 2); CSES: Chronic stress escape strategy; CTC: Circulating tumor cells; Cx43: Connexin 43; Cx32: Connexin 32; dbSNP: Single nucleotide polymorphism database; DEN: Diethylnitrosamine; DNA: Deoxyribonucleic acid; EBV: Epstein-Barr virus; ECM: Extracellular matrix; EMT: Epithelial- mesenchymal transition; GPCR: G protein-coupled receptors; GSK3beta: Glycogen synthase kinase-3beta; GTPase: Small guanosine triphosphateses; HCC: Hepatocellular carcinoma; HPV: Human papilloma virus; ICAM-1: Intracellular adhesion molecule 1; IFN-γ: Macrophage-activating factor; IgE: Immunoglobulin E; LINE-1: Long interspersed element 1; LTR: Long terminal transposanable retroposon; LOX: Lysyl oxidase; MC: Mast cell; MCP-1: Monocyte chemotactic protein; MEIS2: PBX-related homeobox gene MRG1; MET: Mesenchymal-epithelial transition; MHC: Major histocompatibility complex; MMP: Matrix metalloproteinase; NCCCT: Normal cell-cancer cell transition; NF-κB: Nuclear factor kappa-light-chain-enhancer of activated B cells; NOX: NADPH oxidase; PBX1: Pre-B-cell leukemia transcription factor 1; PCN: Pre-cancerous niche; PDX1: Pancreatic homeodomain protein; PI3K: Phosphoinositide-3 kinase; PRR: Pattern recognition receptor; Rac1: Ras-related C3 botulinum toxin substrate 1; RFLP: Restriction fragment length polymorphism; Rho: Ras homolog gene; ROS: Reactive oxygen species; SINE: Short interspersed element; SNP: Single-nucleotide polymorphism; SOD: Superoxide dismutase; TNFα: Tumor necrosis factor alpha; TGFβ: Tumor growth factor beta; TIMP-1: Tissue inhibitor of metalloproteinase 1; TLR: Toll-like receptor; TORC1: Target of rapamycin complex 1; TORC2: Target of rapamycin complex 2; TWIST: Twist-related protein 1; VCAM-1: Vascular cell adhesion molecule; ZEB1: Zinc finger E-box-binding homeobox 1.

## Competing interests

Neither author has a competing interest to disclose.

## Authors’ contributions

This manuscript contains original material that has not been previously published. Both authors equally contributed in thinking, discussing, and writing for the manuscript. Both author read and approved the final manuscript.

## Authors’ information

BB http://www.linkedin.com/in/bruecher.

IS http://www.linkedin.com/pub/ijaz-jamall-ph-d-dabt/1b/69/b92.

## Pre-publication history

The pre-publication history for this paper can be accessed here:

http://www.biomedcentral.com/1471-2407/14/331/prepub
